# Severe Trauma-Induced Coagulopathy: Molecular Mechanisms Underlying Critical Illness

**DOI:** 10.3390/ijms24087118

**Published:** 2023-04-12

**Authors:** Christian Zanza, Tatsiana Romenskaya, Fabrizio Racca, Eduardo Rocca, Fabio Piccolella, Andrea Piccioni, Angela Saviano, George Formenti-Ujlaki, Gabriele Savioli, Francesco Franceschi, Yaroslava Longhitano

**Affiliations:** 1 Department of Anesthesia and Critical Care, AON SS. Antonio e Biagio e Cesare Arrigo, 15121 Alessandria, Italy; christian.zanza@live.it (C.Z.);; 2 Department of Anesthesiology and Perioperative Medicine, University of Pittsburgh, Pittsburgh, PA 15260, USA; 3 Department of Physiology and Pharmacology, Sapienza University of Rome, P. le A. Moro 5, 00185 Rome, Italy; 4 Department of Emergency Medicine, Polyclinic Agostino Gemelli/IRCCS, Catholic University of the Sacred Heart, 00168 Rome, Italy; 5 Department of Surgery, San Carlo Hospital, ASST Santi Paolo and Carlo, 20142 Milan, Italy; 6 Emergency Medicine and Surgery, IRCCS Fondazione Policlinico San Matteo, 27100 Pavia, Italy

**Keywords:** polytrauma physiology, blood coagulation disorders, exsanguination, hemorrhagic shock, hemostasis, trauma

## Abstract

Trauma remains one of the leading causes of death in adults despite the implementation of preventive measures and innovations in trauma systems. The etiology of coagulopathy in trauma patients is multifactorial and related to the kind of injury and nature of resuscitation. Trauma-induced coagulopathy (TIC) is a biochemical response involving dysregulated coagulation, altered fibrinolysis, systemic endothelial dysfunction, platelet dysfunction, and inflammatory responses due to trauma. The aim of this review is to report the pathophysiology, early diagnosis and treatment of TIC. A literature search was performed using different databases to identify relevant studies in indexed scientific journals. We reviewed the main pathophysiological mechanisms involved in the early development of TIC. Diagnostic methods have also been reported which allow early targeted therapy with pharmaceutical hemostatic agents such as TEG-based goal-directed resuscitation and fibrinolysis management. TIC is a result of a complex interaction between different pathophysiological processes. New evidence in the field of trauma immunology can, in part, help explain the intricacy of the processes that occur after trauma. However, although our knowledge of TIC has grown, improving outcomes for trauma patients, many questions still need to be answered by ongoing studies.

## 1. Introduction

Trauma remains one of the leading causes of death in adults despite the implementation of preventive measures and innovations in trauma systems [1]. As a result of growing knowledge about the pathophysiological mechanisms that take place in trauma patients mortality has been significantly lowered, although 25–35% of patients still develop biochemically evident coagulopathy on arrival in emergency departments [2,3,4].

**Figure 1:**
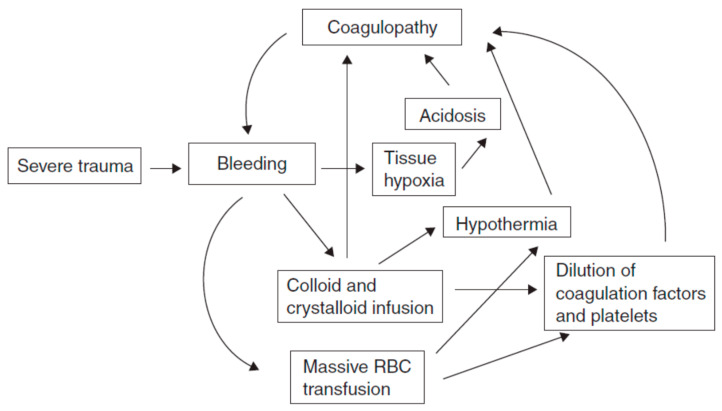
The interplay between metabolic acidosis, hypothermia and progressive coagulopathy in trauma.

**Figure 2:**
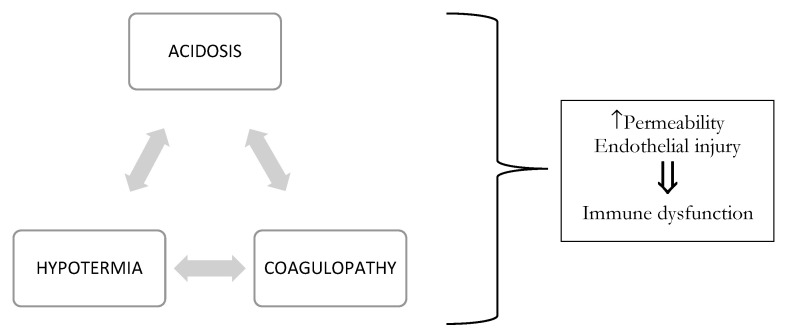
Virchow’s triad that interfere with the clinical course.

The etiology of coagulopathy in the injured patient is multifactorial [2,3], with overlapping contributing factors depending on the injury and nature of resuscitation. Coagulation is the harmonious result of two opposing processes: hemostasis and fibrinolysis, which provide control of bleeding following mild/moderate injury (see Figure 1). In trauma, this balance can be compromised by three main triggers, termed the “vicious triad” or “Virchow’s triad” (see Figure 2) [5,6]: metabolic acidosis (lactic) [7] due to tissue damage, hypothermia due exposure to cold [7,8,9] during the traumatic event and further exacerbated by the administration of cold fluids [10,11], and coagulopathy. These three conditions lead to systemic depletion of coagulation factors, increasing the risk of DIC (disseminated intravascular coagulation) in the first few hours, or later during intensive care.

In the trauma setting, several terms are used in the literature to refer to this condition, including trauma-induced coagulopathy (TIC), acute traumatic coagulopathy (ATC), early-trauma coagulopathy (ECT), and acute coagulopathy of trauma shock(ACoTS) [2,3,12,13,14]. Based on the terminology used by the International Society for Thrombosis and Haemostasis, the term TIC will be used in this review [15].

However, trauma-induced coagulopathy (TIC) is distinct from disseminated intravascular coagulation (DIC), which is defined as “an acquired syndrome characterized by the intravascular activation of coagulation with a loss of localization arising from different causes” [16].

TIC occurs early, long before Virchow’s triad can set in, and is due to a multifactorial biochemical response fueled by dysregulated coagulation, altered fibrinolysis, systemic endothelial dysfunction, inflammatory responses following trauma, and platelet dysfunction. The risk of TIC increases with prolonged hypotension, higher injury severity score, worsening base excess (as compensation of lactate acidosis due to bleeding) and possible concomitant brain injury [17,18], whereas irregular systemic coagulation, often powered by tissue factors on different cell surfaces, is observed in DIC. Eventually, the late pro-thrombotic-antifibrinolytic TIC phenotype mirrors certain DIC phenotypes [19].

The purpose of our narrative review is to survey the most recent literature and present the current understanding of the pathophysiology of trauma-induced coagulopathy, as well as measures for its early diagnosis and treatment.

## 2. Materials and Methods

**Chart 1:**
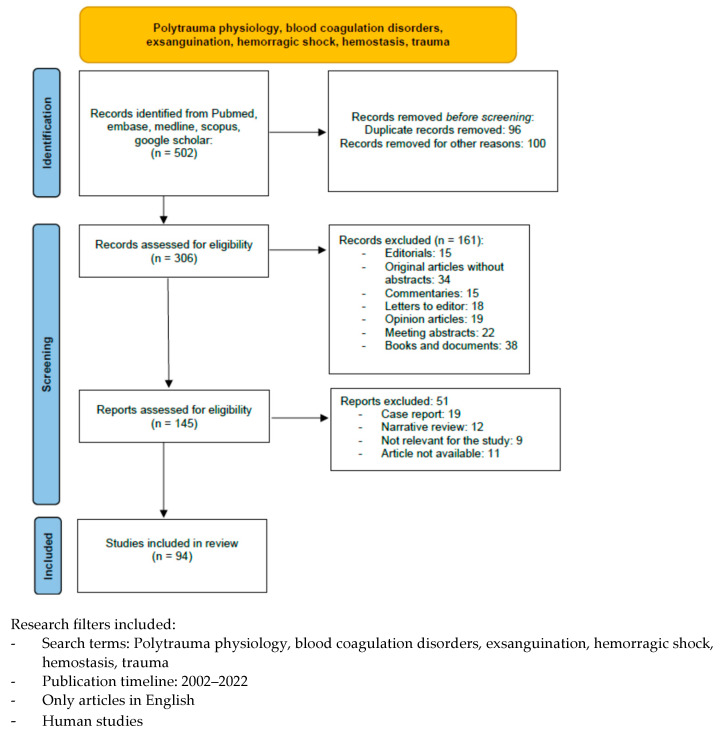
Included and excluded articles.

A literature search was performed using the following databases to identify relevant studies in indexed scientific journals: Pubmed, MEDLINE (via Ovid), EMBASE (via Ovid), and the Cochrane Controlled Clinical trials register, using the following terms: polytrauma physiology, blood coagulation disorders, exsanguination, hemorrhagic shock, hemostasis, and trauma, with filters for humans, language (English), and time of publication (2002–2022). We excluded editorials, commentaries, letters to the editor, opinion articles, meeting abstracts, and original articles lacking an abstract. We identified 502 articles, but only 94 papers were taken into consideration in this review (Chart 1).

## 3. Results

We identified 92 papers of interest to our narrative review. Research was limited to clinical trials, meta-analysis, randomized controlled trials (RCT), review, and systematic review.

In order to be most clearly discuss every aspect regarding this complex disease, we decided to subdivide the discussion into three sub-chapters: TIC pathophysiology, early diagnosis, and treatment.

## 4. Discussion

### 4.1. TIC Pathophysiology

Coagulopathy develops not only when pro-coagulation factors are depleted or diluted, but also when one of the control mechanisms fails. For example the quantity of prothrombin may be insufficient, or its localization within the body may be inappropriate. Trauma is a complex event, so it is a challenge to identify a single mechanism responsible for the development of TIC [20].

TIC can rapidly evolve into different phenotypes, changing from a prevalently anticoagulant to a procoagulant state within hours or days if the patient survives the acute event [15]. Because of the complexity and constantly evolving nature of traumatic injury and TIC, the underlying mechanisms are not fully understood. However, several key mechanisms, including the dysfunction of natural anticoagulant mechanisms [21,22,23], platelet dysfunction [23], fibrinogen consumption and hyperfibrinolysis [22] have been identified as primary components of TIC [24,25].

#### 4.1.1. Inflammatory Responses to Injury

In addition, aPC has anti-inflammatory and cytoprotective properties. In 2012, Cohen MJ et al. published a study of 203 patients with severe trauma. The results showed that early coagulopathy in this type of patient was related to high aPC levels and, subsequently, protein C depletion as early as six hours after injury [26]. Patients with protein C depletion had a higher risk of developing acute lung injury, ventilation-associated pneumonia, multi-organ failure, and death [27]. In the past, experiments have been conducted to understand the role of these mechanisms [28]. Selective antibody-mediated inhibition of the anticoagulant function of aPC reduced the rate of coagulopathy but not mortality, whereas the inhibition of anticoagulant and cytoprotective functions increased mortality after a challenge with trauma/hemorrhagic shock [29,30,31,32,33] or lipopolysaccharide injection [29]. Cytoprotective functions of aPC may also play a role in the endothelial barrier function of pulmonary capillaries, as suggested by in vitro studies [30,31] and human studies associating persistently low levels of protein C in severely traumatized and mechanically ventilated patients with increased rates of pneumonia [32].

#### 4.1.2. Systemic Endothelial Dysfunction

Data from various studies have demonstrated that shock is the key factor in the development of TIC. There is degradation of the endothelial glycocalyx, a protective stratum, during an injury, leading to a systemic release of syndecan-1, a degradation product of the glycocalyx [33,34]. Increased syndecan-1 levels are also associated with worse mortality outcomes [35].

The release of endogenous heparan sulfates from the glycocalyx may also result in self-anticoagulation by increased circulating endogenous heparinoids [20].

In addition, tissue hypoperfusion also releases damage-associated molecular patterns (DAMPs), activates the contact pathway, and induces the expression of thrombomodulin and endothelial protein C receptor (EPCR) on the endothelial surface to activate protein C. The strong activation and consumption of protein C may deplete protein C stores, potentially leading to a reduction in endothelial-protective signaling through the aPC receptors, protease-activated receptor-1 (PAR-1) and endothelial protein C receptor (EPCR), independently of the role of aPC as an anticoagulant, potentially exacerbating endothelial dysfunction [36]. In 2007, Johansson et al. conducted a prospective study and observed that protein C depletion in trauma patients correlated with elevated markers of endothelial damage and coagulopathy and a three-fold increased risk of mortality [21].

Furthermore, the release of microparticles and (DAMPs) has been demonstrated to occur following direct tissue damage. Studies have shown that direct damage prompts the release of thrombin-rich microparticles into the systemic circulation. The local effects of these may contribute to hemostasis, whereas wider systemic release may lead to a DIC-like phenotype and resultant coagulopathy. In the prospective observational PROMMTT study conducted by Mtijevic et al., 180 trauma patients were observed [37]. In all of these patients, elevated levels of endothelium-derived microparticles, erythrocytes, and leukocytes were found in the circulation compared with the control group. Interestingly, high levels of microparticles were also found in traumatic brain injury patients [38].

In contrast, it has been observed that DAMPs are released because of both the active process induced by hypoperfusive tissue hypoxia, and the passive process as the result of cell lysis, and can directly stimulate a sterile immune or inflammatory response [39,40]. Persistent release of DAMPs can power or maintain hyperactivation of the immune system during the active phase, which will lead to a pathway to immune dysfunction, essentially forming a feedback loop of tissue damage leading to inflammation, which in turn leads to further tissue damage and cellular dysfunction. This process of immune dysfunction following traumatic injury is dominated by the pattern recognition receptors TLR2, TLR4 and TLR9.

#### 4.1.3. Dysregulated Coagulation

In normal conditions, the coagulation process following injury involves increased thrombin production, fibrin deposition, and clot formation through the extrinsic pathway. Instead, systemic coagulation triggered by thrombin from the injury site is inhibited by circulating antithrombin III, or by the binding of thrombin to constitutively expressed thrombomodulin on nearby undamaged endothelial cells [41]. Protein C, a systemic anticoagulant, is converted to activated protein C (aPC) by the thrombin complex with thrombomodulin. aPC is a serine protease that inactivates factors Va and VIIIa and eliminates plasminogen inhibitors [33]. Thus, aPC may play a protective function by inhibiting thrombosis during periods of reduced flow. In trauma patients with hypoxic injury, a correlation has been observed between TIC and elevated aPC level, reduced levels of non-activated protein C, and elevated soluble thrombomodulin [31].

#### 4.1.4. Fibrinogen Depletion and Alterations in Fibrinolysis

Fibrinogen is the most abundant coagulation factor in blood, with circulating levels in the range of 2–4 g/L in a healthy adult and a circulating half-life of ~4 days [42]. Conversion of fibrinogen to fibrin occurs via thrombin-mediated cleavage at two sites, exposing binding sites for other fibrin molecules, thereby giving rise to spontaneous polymerization. However, despite a high concentration of fibrinogen in healthy blood, it is the first clotting factor and has the lowest concentration among clotting factors in patients with massive bleeding [43]. It has long been known that trauma and hemorrhagic shock are associated with a hyperfibrinolytic state, occuring in the first few minutes and sometimes persisting for hours after injury [44]. This is due to hypoperfusion-related hypoxia combined with the negative feedback associated with prothrombin generation stimulating the endothelial release of tissue plasminogen activator (tPA). The consumption of endogenous plasminogen activator inhibitor-1 (PAI-1) by TIC-mediated aPC further compromises the fibrinolytic balance, leading to uninhibited tPA-mediated conversion of plasminogen to plasmin [45]. The transformation of thrombin to protein C activation can also reduce thrombin-activated fibrinolysis inhibitor (TAFI) activation, further reinforcing fibrinolytic activity [46]. These mechanisms lead to the hyperfibrinolysis observed in trauma patients with TIC, resulting in increased tPA levels, decreased PAI-1, and increased D-dimer [17]. Indeed, it has been shown that the lowest levels of fibrinogen may be found in patients admitted to the ICU [47]. Moreover, fibrinogen is also an important marker of the need for transfusion at 24 h and mortality at 28 days [48,49].

#### 4.1.5. Platelet Dysfunction

Plates play a crucial role in hemostasis after injury [50] while also regulating endothelial homeostasis and the immune response [51,52]. Failure of these mechanisms contributes to the development of TIC in more than 50% of trauma patients with major injuries [53]. Furthermore, platelet count has been shown to be inversely correlated with the need for transfusion and early mortality [54]. Interestingly, in trauma patients, there is often a phenomenon of ‘platelet exhaustion’ characterized by an altered platelet aggregation response, despite the fact that the platelet count is in range [55,56]. Specifically, following major trauma and subsequent shock, there is a release of TF (tissue factor), platelet-activating factor and vWF [57], leading to platelet over-activation, thus creating a pool of activated platelets in the circulation that are ‘depleted’ or exhausted following the release of their procoagulant and anticoagulant factors. These platelets are rendered useless in terms of primary hemostasis. In addition, polytrauma patients have increased sensitivity to tPA-mediated fibrinolysis due to reduced platelet PAI-1 release [58]. Some recent studies have reported that there are several platelet phenotypes involved in platelet dysfunction in TIC. Primary platelet effects contribute to early TIC and, more importantly, to hemorrhage. In contrast, the immunoregulation carried out by platelets contributes to the subsequent hypercoagulability of TIC [59,60]. In 2018, a study performed by Zipperle J. et al. showed that traumatic injury stimulates platelet activation, which in turn promotes platelet–leukocyte binding, creating platelet–leukocyte aggregates. These aggregates stimulate the systemic release of platelet factor 4 and the expression of TF, fibrinogen, and factor Xa, leading to the pro-coagulant state [61].

### 4.2. Diagnosis

All trauma patients undergo routine laboratory tests such as complete blood count, serum electrolytes, arterial blood gas analysis, and standard coagulation tests. These tests allow early detectection of Virchow’s triad: acidosis, hemodilution, and severity of shock (base deficit and/or serum lactate level).

**Figure 3:**
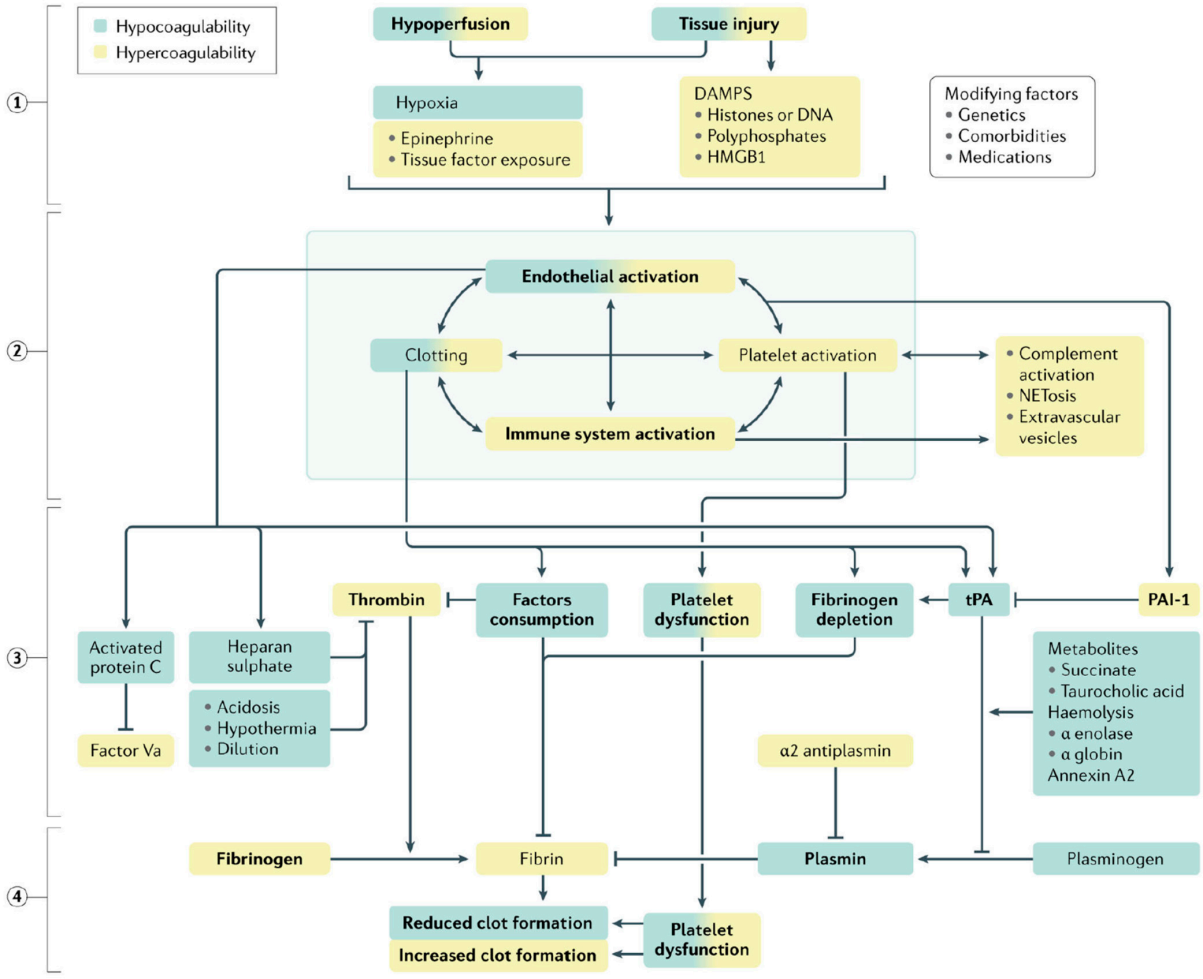
Qualitative changes in coagulation parameters in trauma-induced coagulopathy. Tissue injury and shock ① synergistically activate the endothelium, platelets and the immune system ② to generate an array of mediators that reduce fibrinogen, impair platelet function and compromise thrombin generation ③, ultimately resulting in inadequate clot formation for hemostasis ④. Increased fibrinolysis via plasmin generation further compromises hemostatic capacity. These deficits are accentuated by ongoing blood loss, hemodilution, metabolic acidosis and hypothermia. A colour gradient indicates that the mechanism can result in both hypocoagulation (green) and hypercoagulation (yellow). DAMPs, damage-associated molecular patterns; HMGB1, high mobility group protein B1; PAI-1, plasminogen activator inhibitor-1; tPA, tissue plasminogen factor [62].

Easy-to-perform coagulation tests such as prothrombin time, international normalized ratio and activated partial thromboplastin time are standard-of-care in the definitive diagnosis of coagulopathy. In addition, fibrinogen and D-dimer levels are also measured. The latter serve as surrogate markers of coagulation factor consumption and hyperfibrinolysis (see Figure 3).

The high levels of D-dimer, a fibrin degradation product, have been associated with the severity of tissue damage and the state of hyperfibrinolysis, and therefore also fibrinogen depletion, especially in the acute phase [62]. The results of a multicenter retrospective study involving 519 adult trauma patients was published in 2016 showed that patients with high D-dimer and low fibrinogen had the highest mortality compared with control groups. In 2019, TACTIC, a prospective multicenter observational cohort study of 940 patients, reported a 7-fold higher DD level (died vs. survived: 103,170 vs. 13,672 ng/mL, *p* < 0.001) [63] in the group of severely injured patients with a preponderance for traumatic brain injury.

The quantity of fibrinogen is equally important, since acquired deficiency leads to the worsening of hemorrhaging and increased mortality [64]. Therefore, it is necessary to promptly correct the deficiency of this factor with cryoprecipitate or fibrinogen concentrates on obtained laboratory findings and/or TEG/ROTEM results.

Although coagulation factors are not commonly tested in injured patients, a deficiency of such factors following hemodilution and/or transfusion of blood products may exacerbate TIC. In addition to fibrinogen, described above, two other factors, V and VIII, are the most labile and may be selectively diminished during trauma resuscitation, particularly when the proportion of units transfused is not calibrated. Fibrinogen, thrombin, Factor V, Factor VIII, Factor IX, Factor X, and activated Protein C levels are negative predictors of TIC adjusted for age, injury, and shock [65].

**Figure 4:**
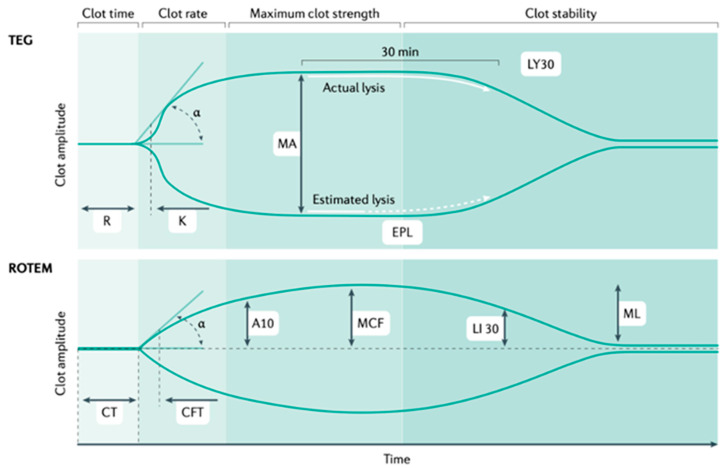
TEG (thromboelastography) and ROTEM (rotational thromboelastometry).

The PROMMTT study involving 165 severe trauma patients demonstrated deficits in laboratory results, and biomedical research has led to the development of laboratory tests such as TEG (Thromboelastography) and rotational thromboelastometry (ROTEM), which are widely used viscoelastic assays employed to assess and manage TIC [66,67] (see Figure 4). Both techniques help to identify alterations in normal fibrinolysis early in severe trauma, allowing rapid diagnosis and early treatment of acute hemorrhage.

TEG and ROTEM measure the speed of thrombin generation as measured by clot firmness of 2 mm (reaction time (R) in TEG and clotting time (CT) in ROTEM); the speed of clot formation, which includes the contribution of fibrinogen (α angle in both assays); the maximum clot strength (maximal amplitude (MA) in TEG and maximal clot firmness (MCF) in ROTEM); and the magnitude of fibrinolysis (LY30; that is, the percentage reduction in the area under the curve at 30 min after MA in TEG, and LY30, the residual clot firmness at 30 min after CT in ROTEM) (see Figure 4) [20]. TEG stands for thromboelastography; ROTEM stands for rotational thromboelastometry; MA stands for maximal amplitude; MCF stands for maximal clot firmness; and LY30- stands for the amplitude at 30 min.

### 4.3. Treatment

**Figure 5:**
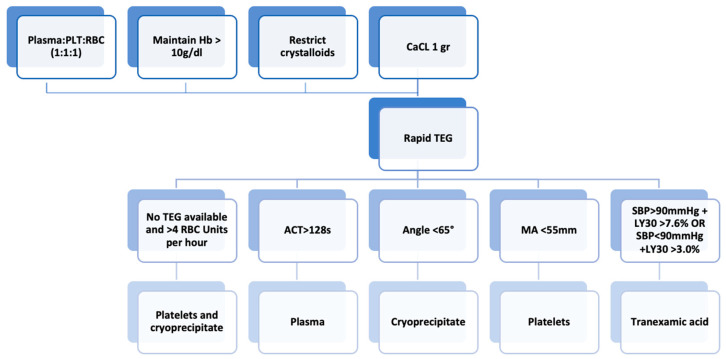
Subsequent management of trauma-induced coagulopathy. Abbreviations: ACT, activated clotting time; aPTT, activated partial thromboplastin time; CCA, conventional coagulation assay; LY30, percentage reduction in the area under the curve at 30 min after MA in TEG; MA, maximum amplitude; PT, prothrombin time; RBC, red blood cell; SBP, systolic blood pressure; VHA, viscoelastic hemostatic assay.

The early recognition and prompt management of TIC (see Figure 5) can reduce mortality in patients with severe injuries from trauma. Over time, increased understanding of the pathophysiology of TIC and biotechnological and therapeutic innovations have allowed rapid intervention and a reduction in short- and long-term complications. Damage control resuscitation consists of permissive hypotension, the avoidance of excessive crystalloids, hypothermia, and acidosis, rapid surgical correction of hemorrhages, and early transfusion.

The goal of any hemostatic therapy is to minimize blood loss and transfusion requirements; increased transfusion demands are recognized to increase the morbidity and mortality in trauma patients. Among patients with similar injury severity scores (ISS), mortality is about four times higher in case of coagulopathy Massive hemorrhage and massive transfusion in patients with multiple injuries are associated with impaired coagulation. Indeed, sufficient thrombin and coagulation substrates are required to achieve adequate hemostasis. In addition to platelets, on whose surface most thrombin is formed, fibrinogen can be considered a primary coagulation substrate. If sufficient thrombin is produced, it transforms fibrinogen into stable fibrin, which leads to the clot compactness that develops in the presence of factor XIII.

It is important to maintain euvolemia after a traumatic injury and massive hemorrhage to prevent the development of shock and acidosis, which are directly related to coagulopathy and worse outcomes. The optimal choice of volume expanding fluid remains controversial in this context [17]. Crystalloids impair the coagulation system, mostly due to their diluting effect. Ringer’s lactate fluid therapy reduces indices of tissue hypoxia but does not influence the alterations in fibrinogen metabolism resulting from hemorrhage [68]. In adults, a bolus of 1 L of isotonic solution may be necessary to obtain a congruous response. If the patient does not respond to the initial bolus of crystalloids, then hemotransfusion should be started. Gelatin solutions also cause a diluting effect while compromising fibrin polymerization. They can also lead to a decrease in clot elasticity, and a reduction in clot weight [69]. Hydroxyethyl starch solutions (HES) can increase hemorrhagic tendencies. HES causes hypocalcemia, inhibition of the fibrinogen receptor (GPIIb-IIIa), and a disorder of fibrin polymerization that can overcome the anticoagulant effect of gelatin [70].

Tranexamic acid (TXA) is the most widely studied antifibrinolytic agent in the trauma setting. A lysine analog that blocks the lysine binding site on the fibrinolytic enzyme plasmin, it is essential for plasmin binding to fibrin. Thus, the regular plasmin effect, fibrinolysis of the blood clot, is blocked. At low doses, tranexamic acid acts as a competitive inhibitor of plasmin, while at high doses it is a non-competitive inhibitor.

The studies reported below identify a subgroup of trauma patients who are expected to benefit from timely and proper treatment with TXA. In 2011, the results of the CRASH-2 study were published reporting reduced all-cause mortality in adult trauma patients treated with tranexamic acid [71]. Thereafter, this study demonstrated that TXA, when administered more than 3 h after the acute event, leads to an increased risk of venous thromboembolism [71,72] and risk of hemorrhage-related death [73]. International guidelines support the hypothesis that TXA should be administered early, within 3 h after a traumatic event [74].

A recent meta-analysis evaluated the incidence of thrombotic events associated with the administration of TXA to patients with trauma-induced hemorrhage, as well as surgical and medical hemorrhage, and found no significant increase in thrombotic events associated with any dose of TXA [75]. However, patients with the more common fibrinolytic ‘shutdown’ phenotype seem to have a higher risk of thromboembolic complications and long-term organ failure, and therefore may be at increased risk of damage from TXA treatment. Therefore, in clinical settings where immediate access to TEG allows the early determination of the fibrinolytic phenotype, it is strongly recommended that an empirical bolus of TXA be administered as soon as possible (within three hours of injury) and that TEG be used to assess whether additional doses of TXA are required.

The current guidelines recommend administering an initial 1 g bolus of tranexamic acid infused over 10 min at the trauma scene, followed by an infusion over 8 h.

#### 4.3.1. Goal-Directed Transfusion

Massive blood transfusion is one of the primary strategies in the treatment of hemorrhagic shock in trauma patients. Blood product transfusions are known to improve tissue perfusion and increase oxygen transport capacity to tissues. However, despite this advantage, it has been shown that transfusions can trigger or worsen TIC. Massive transfusion protocols vary among different centers around the world, and ratios of plasma to packed red blood cells range from 1:1 to 1:10. Three clinical trials (PROMMTT [76], PROPPR [77] and COMBAT [78]) have shown that patients at high risk of developing TIC can benefit in terms of survival from transfusion a protocol involving plasma, packed red blood cells, and platelets in equal ratios (1:1:1).

Notably, in the PROMMTT (PRospective, Observational, Multicenter, Major Trauma Transfusion) study, the authors found that “early” transfusion of plasma was associated with reduced 24 h (odds ratio [OR] 0.47, 95% CI 0.27–0.84) and 30-day (OR 0.44, 95% CI 0.27–0.73) mortality compared with patients who received lower plasma/RBC ratios or who did not receive early plasma but “caught up” to ratios approaching 1:1 by 24 h [79].

The PROPPR study published in 2015 reported no difference in survival at 24 h and 30 days in two groups of patients (1:1:1 ratio versus a 1:1:2 ratio of plasma to platelets to RBCs in a transfusion). However, the first group (1:1:1 ratio) was less likely to die from a hemorrhage [77].

#### 4.3.2. Thromboelastography-Based Transfusion

The fast and simple method of thromboelastography (TEG) may help to predict the need for blood transfusions in severely injured trauma patients. Going forward, resuscitation protocols guided by the results obtained from TEG may become the gold standard. In 2016, a prospective randomized controlled trial conducted by Gonzalez et al. reported lower mortality inpatients transfused based on the resultsof TEG with results obtained from laboratory testing [80] (19.6% vs. 36.4%, respectively).

#### 4.3.3. Fibrinogen Concentrates

Fibrinogen plays an important role in hemostasis during the early phases of trauma, and low fibrinogen levels after severe trauma are associated with hemostatic impairment, massive bleeding, and poor outcomes. Early administration leads to the prevention of TIC and consequently, less need for blood product transfusions as demonstrated in the randomized controlled “FiiRST” (Fibrinogen in the initial resuscitation of severe trauma) trial [81] published in 2016 in which the timely infusion of concentrated fibrinogen was shown to reduce TIC-related complications by maintaining a high fibrinogen level in trauma patients. Current guidelines recommend fibrinogen replacement during major bleeding when fibrinogen levels drop below 1.5 mg/mL. Cryoprecipitate, a pooled blood product derived from fresh frozen plasma, is commonly used to increase fibrinogen levels in the acute setting.

#### 4.3.4. Pharmaceutical Hemostatic Agents

The available hemostatic drugs used as adjuvants for the management of severe coagulopathy in the bleeding patient include recombinant factor VIIa, prothrombin complex concentrate, antifibrinolytic agents and desmopressin.

While recombinant human factor VIIa is an adjunctive treatment for trauma-associated coagulopathy, it should be reserved for life-saving treatment. When used, it is important to first correct acidosis, hypothermia, thrombocytopenia, and hypofibrinogenemia The rationale for use is that this factor binds to the tissue factor exposed as a result of trauma. The complex that is subsequently created stimulates clot formation at the site [82].

Prothrombin complex concentrate (PCC) is a concentrate enriched in factors II, VII, IX and X. Only a few trauma centers have used PCC in the treatment of TIC [83]; the results obtained seem excellent but further clinical studies are needed to better understand the mechanism of activity. The clinical use of PCC has been widely studied in counteracting warfarin-induced anticoagulation, as PCC contains vitamin K-dependent clotting factors [84].

Coagulopathy is routinely corrected with the use of fresh-frozen plasma (FFP), because it provides volume support to reverse hypoperfusion and also replaces coagulation factors [85,86].

Tissue hypoperfusion occurring in the first moments in trauma patients is one of the main factors responsible for the development of coagulopathy. To counteract this mechanism, resuscitation with high volumes of crystalloids is used; however, this further worsens the coagulopathy due to the dilution of clotting factors. Moreover, the severity of tissue injury is associated with the consumption of clotting factors, which leads to further worsening of coagulopathy. Studies in the past have recommended the use of FFP in the treatment of TIC, since FFP not only provides the necessary volemic support but also coagulation factors [87,88]. Unfortunately, FFP is not so readily available, since it requires cross-matching and thawing before administration.

FFP therapy is also associated with a delay in the reversal of coagulopathy [85,86]. Recently, there has been increased use of PCCs as an alternative to fresh-frozen plasma. It has been observed that the administration of PCC in a concentrate form provides an advantage in overcoming early coagulopathic effects of large-volume fluid resuscitation in trauma patients [85,86]. The major concerns about the clinical use of PCC have been the development of thromboembolic complications and the higher cost of therapy compared to FFP [85,86].

In the retrospective analysis of a prospectively maintained database of all coagulopathic (INR ≥ 1.5) trauma patients presenting to level I trauma centers, conducted by Joseph B. et al. [89], the effect of PCC + FFP therapy (*n* = 63 patients) vs. FFP alone (*n* = 189 patients) was studied. The results revealed that use of PCC helps to restore INR to normal values faster (394 vs. 1050 min; *p* = 0.001), leading to less demand for concentrated blood units (6.6 vs. 10 units; *p* = 0.001) and, most importantly, reduced mortality rates (23 vs. 28%; *p* = 0.04). In fact, the authors demonstrated a survival difference in patients who received PCC + FFP therapy compared to patients who received FFP therapy alone. This difference in mortality may be attributed to the rapid reversal of coagulopathy and factor replacement in patients who received PCC together with FFP [89].

There is insufficient clinical evidence regarding desmopressin to support its use in the trauma population, except in patients with preexisting bleeding diathesis [90]. Preliminary results from animal studies conducted in 2008 and 2010 showed an improvement, but not complete correction of platelet dysfunction induced by hypothermia or acidosis [91,92,93].

Instead, ketamine can prevent indirectly acidosis because acts both as sedative and good inotropic on trauma, but its effect is very unclear in trauma induced coagulopathy. If on the one hand some studies demonstrate an inhibitory activity on platelets on the other hand it is an excellent drug for maintaining good hemodynamics and indirectly avoiding acidosis which in turn inhibits platelet function [94].

## 5. Conclusions

As described in this narrative review, TIC is a result of a complex mechanism of interactions of different pathophysiological processes such as altered coagulation, inflammation, and cellular dysfunction. New evidence in the field of trauma immunology can partly explain the complexity of the processes that occur after trauma. However, even though our knowledge of TIC has increased, improving outcomes of trauma patients, many questions remain that will hopefully be clearly answered by ongoing and future studies.

## 6. Future Perspectives

Ongoing trials are currently investigating the early use of fibrinogen or cryoprecipitate and the administration of specific coagulation factors to correct TIC. Further investigations are needed into the role of inflammation and the mechanisms of hypercoagulability and organ dysfunction during late TIC. A better understanding of the complex and dynamic pathophysiology of TIC may help to reduce preventable deaths due to trauma-induced shock and organ dysfunction in the future.
